# Explaining Willingness to Pay for Andean Grains Through Health Consciousness

**DOI:** 10.3390/foods15122195

**Published:** 2026-06-18

**Authors:** Elizabeth Emperatriz García-Salirrosas, Karla Liliana Haro-Zea, Ángel Acevedo-Duque, Elena Matilde Urraca-Vergara, Amit Roy Flores Rivera, Dany Yudet Millones-Liza

**Affiliations:** 1 Faculty of Management Science, Universidad Autónoma del Perú, Lima 15842, Peru; egarciasa@autonoma.edu.pe; 2 Universidad Autónoma de Baja California, Facultad de Ciencias de la Ingeniería, Administrativas y Sociales, Campus Tecate, Tecate 21460, Mexico; kharozea@gmail.com; 3 Grupo de Investigación de Estudios Organizacionales Sostenibles, Universidad Autónoma de Chile, Santiago 7500912, Chile; angel.acevedo@uautonoma.cl; 4 Programa de Estudios de Ingeniería Industrial, Facultad de Ingeniería, Universidad Privada Antenor Orrego, La Libertad 13000, Peru; eurracav@upao.edu.pe; 5 Escuela Profesional de Administración de Turismo Sostenible y Hotelería, Facultad de Ingeniería y Gestión, Universidad Nacional Autónoma de Huanta, Huanta 05121, Peru; aflores@unah.edu.pe; 6 Universidad Tecnológica del Perú, Lima 15046, Peru

**Keywords:** health consciousness, willingness to pay, Andean grains, theory of planned behaviour, self-identity, perceived behavioural control, sustainable food choice, university students

## Abstract

This study examines the relationship between health consciousness, the components of the Theory of Planned Behaviour (TPB) and self-identity, as well as consumers’ willingness to pay more for Andean grains, which are recognised for their nutritional benefits and contribution to sustainable food systems. The study was conducted with 600 young university students living in Lima, Peru. Using Partial Least Squares Structural Equation Modelling (PLS-SEM), the findings revealed a positive association between health consciousness and attitude, subjective norms, perceived behavioural control, and self-identity related to healthy food consumption. Of the TPB components, self-identity was found to be the strongest predictor of willingness to pay more for Andean grains, followed by perceived behavioural control; attitude and subjective norms, however, showed no significant effect. These results suggest that the willingness to pay for sustainable heritage foods is driven more by identity-based motivations and perceived access conditions than by favourable evaluations or social pressure alone. The study extends the TPB by incorporating health consciousness as an antecedent variable and by highlighting the prominent role of self-identity in sustainable food choices within the context of an emerging economy. Furthermore, the findings provide practical insights for designing marketing strategies and public policies aimed at promoting healthy and sustainable food consumption.

## 1. Introduction

The transition towards sustainable food systems aims to increase sustainability worldwide by addressing the mechanisms that support biodiversity conservation, food security, and public health [1]. Some recent studies have focused on analysing strategies that aim to reduce food waste and promote the reuse of aesthetically imperfect food products through circular economy practices [2]. In addition to environmental and economic factors, the concept of sustainable food systems increasingly emphasises the importance of consumer health and dietary behaviour for achieving long-term sustainability goals. In this regard, food choices are associated not only with ecological responsibility, but also with individual motivations relating to well-being and healthy lifestyles. In this context, sustainable food systems promote health awareness by prioritising the nutritional quality of diets and encouraging the consumption of nutritious, safe, and healthy foods [3]; health education involves raising public awareness of the benefits of sustainable diets. Educational and health institutions play a key role in fostering healthy eating habits and encouraging behavioural change through awareness campaigns that promote healthier lifestyles [4,5].

Within this framework, health consciousness has been identified as having a positive influence on the purchase of ecological and sustainable products. This means that consumers who are more health-conscious are more likely to adopt sustainable dietary practices, provided they trust the integrity of the products. This motivates them to explore and select healthier food options [6,7]. Consequently, health consciousness has emerged as a robust predictor of changes in food consumption patterns. However, such effects are moderated by emotional and social motivations [8,9]. Therefore, it is essential to understand how health consciousness interacts with psychological and behavioural mechanisms in order to explain sustainable food choices in emerging markets. This is a significant turning point in our understanding of how individuals make food decisions in contexts where health and sustainability converge. Health consciousness does not operate in isolation; rather, it interacts with cognitive and social constructs such as self-identity, attitudes, subjective norms, and perceived behavioural control, which mediate its translation into concrete consumption behaviours [10,11].

In Latin America specifically, food practices are deeply rooted in cultural and social traditions, while populations simultaneously face economic inequalities that significantly impact consumption patterns. Urban populations tend to consume a more diverse range of foods, whereas rural populations depend more heavily on staple foods [12,13]. On the other hand, previous studies suggest that greater nutritional knowledge can influence food choices, even if it does not necessarily lead to healthy behaviours due to external factors such as accessibility and cost [14,15]. For example, although university students often have knowledge about food, they face multiple barriers such as lack of time, academic stress, social influence, demanding academic routines, and financial constraints, which all limit their consumption of sustainable food [16,17,18]. These contextual conditions are particularly relevant in emerging economies, where the choice of healthy and sustainable food is not solely determined by nutritional awareness, but also by psychological, cultural, and perceived control factors that influence consumer decision-making. Therefore, the mechanism by which health awareness interacts with the dimensions of the TPB model to influence the willingness to pay for Andean grains remains unclear in the Latin American context, where cultural and identity factors intertwine with nutritional motivations.

Andean grains are notable as sustainable foods due to their high nutritional value, ability to withstand adverse climatic conditions, and low environmental impact. Their properties generate significant health benefits, and as research has progressed, they have become key ingredients in functional foods [19,20]. In addition, their low input requirements for water and fertilisers mean they have a low environmental footprint and contribute to biodiversity conservation [21,22]. These products have gained global attention, particularly in high-income countries, where consumers are seeking health benefits and cardiovascular protection, as well as environmentally friendly products [23,24]. Despite these benefits, consumption of these grains remains minimal or nonexistent in countries or among populations with lower income levels. This creates a relevant paradox: Peru, the country of origin of these grains and custodian of their genetic and cultural diversity, faces a situation in which its own consumers show low levels of consumption. This disconnection cannot be explained solely by economic constraints [11,25].

In this context, previous research involving Peruvian consumers has shown that even among health-conscious individuals, willingness to pay for Andean grains is influenced by deeper psychological factors such as self-identity, linked to healthy habits and perceived control over one’s eating behaviour. Social pressure and favourable attitudes towards the product are insufficient to prompt a purchase. This suggests that consumers may value Andean grains not only because they have a positive perception of them, but also because these foods align with their personal identity and lifestyle aspirations. In contexts where food products have cultural, symbolic, and health-related significance, identity-based motivations may therefore have a stronger influence on economic decisions than traditional attitudinal evaluations or perceived social expectations. This evidence reveals a theoretical and empirical gap. While the literature shows that self-identity and perceived control influence willingness to pay, there is no known integrative mechanism through which health consciousness activates the components of the Theory of Planned Behaviour (TPB) and subsequently translates into willingness to pay. Specifically, this study aimed to identify the extent to which health consciousness modifies the effect on the willingness to pay more for Andean grains among urban consumers in Peru. However, there is a lack of evidence regarding the interaction of the constructs.

Unlike previous studies, this research seeks to expand the explanatory power of the TPB in sustainable food consumption contexts within emerging economies by incorporating health consciousness as an external antecedent. Specifically, the study proposes that health consciousness acts as a psychological driver that can simultaneously activate the cognitive, social, and control dimensions of the TPB, thereby shaping the willingness to pay for sustainable heritage foods. Thus, the study moves beyond the TPB’s traditional focus on behavioural intention by examining how health-oriented motivations translate into economic outcomes such as willingness to pay. The study contributes to the current debate about the hierarchy of TPB components by conducting an empirical analysis of the role of self-identity in willingness to pay compared to attitude and subjective norms. The analysis specifically focuses on evaluating the consumption of a product that embodies cultural and heritage value.

Similarly, this study aims to provide one of the first pieces of evidence on how health consciousness shapes the willingness of Peruvian consumers to pay for Andean grains through the four TPB components simultaneously, thereby identifying which constructs activate or fail to activate this willingness. Unlike behavioural intention, which reflects a general predisposition towards performing a behaviour, willingness to pay represents a more concrete economic commitment involving acceptance of a premium price for a product. Therefore, examining willingness to pay provides a more precise understanding of how psychological and health-related motivations translate into consumer valuation within sustainable food markets. Within this framework, the study examines the effect of health consciousness on the willingness to pay for Andean grains in Peru, as well as the mediating role of self-identity, attitude, subjective norm, and perceived behavioural control within the TPB.

## 2. Review of the Literature and Development of Hypotheses

### 2.1. Theory of Planned Behaviour

The Theory of Planned Behaviour (TPB), developed by Icek Ajzen, is a widely used psychological model to predict and explain human behaviour in various contexts. Examples include intentions to be physically active, energy-saving behaviour, blood donation intentions, and technology adoption [26,27,28,29]. This theory extends the Theory of Reasoned Action by incorporating the concept of perceived behavioural control, thereby enabling the analysis of behaviours that are not entirely voluntary [30,31].

Its main components are:

- Attitude towards behaviour, which refers to the positive or negative evaluation of a specific action;
- Subjective norms, which are based on the importance individuals assign to others’ opinions regarding their behaviour; and
- Perceived behavioural control [32]. Together, these factors determine behavioural intention, which, according to previous studies, is the most immediate predictor of actual behaviour. In the present study, the Theory of Planned Behaviour (TPB) provides the structural framework for examining how health consciousness is translated into willingness to pay for Andean grains among Peruvian university consumers through attitude, subjective norms, perceived behavioural control, and self-identity.

### 2.2. Health Consciousness

Health consciousness refers to individuals’ understanding and knowledge of health-related issues, including disease prevention and healthy behaviours. It plays a fundamental role in promoting healthy lifestyles [33]. Factors that promote health awareness include:

- Individual factors: self-esteem, health literacy and knowledge;
- Social factors: social support and awareness campaigns;
- Contextual factors: educational environments and public policies;
- Psychological factors: fear and risk perception [34,35]. Given its importance, this study incorporates health consciousness as an exogenous variable within the TPB framework to determine its role in motivating intentions and consumption behaviours, particularly in contexts where healthy foods, such as Andean grains, represent both a nutritious choice and a sustainable option.

### 2.3. Sustainable Foods

Sustainable foods promote human health, have a low environmental impact, and are culturally acceptable. In other words, they are foods that ensure global food security without compromising natural resources for future generations [36,37]. Examples include plant-based foods such as fruit, vegetables, and nuts [38].

Factors that facilitate their consumption include environmental awareness and education, accessibility, availability, and price [39,40,41]. Thus, every consumer decision to choose such foods promotes production practices oriented toward sustainability, generating a multiplier effect that transcends individual choice and contributes collectively to the transformation of food systems [42].

In this context, Andean grains are a prime example of sustainable food, meeting all three of the defining criteria of this category: (1) they have a high nutritional value that is internationally recognised; (2) they have a low environmental impact due to their limited water and fertiliser requirements; and (3) they are deeply culturally accepted within the Andean context [20,43].

### 2.4. Hypothesis Development

Health consciousness is an important motivator for promoting healthy behaviours and self-perception. According to the literature, health-related self-identity directly influences health consciousness and consumer attitudes. Furthermore, it has been demonstrated that health consciousness does not operate in isolation, but rather makes individuals more aware of the expectations of their social environment with regard to healthy eating, thereby increasing their perception of approval or disapproval of their food choices [44,45]. In this sense, individuals who are more health-conscious are more likely to internalise socially shared beliefs about healthy eating and pay more attention to the expectations of family members, peers, and social groups regarding this issue. As healthy lifestyles become more socially accepted, health-conscious consumers may feel more pressure to make food choices that align with health and sustainability values.

Other studies state that health consciousness is key to understanding how consumers adopt products such as Andean grains. These grains not only satisfy nutritional needs, but also symbolise a healthy and sustainable lifestyle [46,47]. Similarly, previous research suggests that health consciousness is linked to positive attitudes towards healthy foods. For example, health consciousness improves attitudes towards cereals and organic grains, which increases the intention to purchase these products [48,49].

Similarly, the perceived benefits of Andean grains reinforce their appeal among health-conscious consumers, who have a positive attitude towards consuming them. It is well established that such consumers develop favourable attitudes towards healthy food [50,51]. Furthermore, health consciousness has been conceptualised as a multidimensional construct encompassing the perception, knowledge, and motivation required to adopt health-promoting behaviours [52,53]. In addition to influencing favourable evaluations, health consciousness may also enhance perceived behavioural control, as consumers who are more informed and motivated regarding health tend to view themselves as more capable of making consistent dietary decisions, even in situations involving obstacles such as higher prices, limited accessibility, or restricted availability. In this regard, knowledge of the nutritional benefits of food and confidence in one’s ability to maintain healthy eating habits can reinforce the perception of control over food-related decisions.

Therefore, greater health consciousness is associated with favourable attitudes towards healthy foods, stronger subjective norms reflected in social approval, and higher perceived behavioural control, which refers to confidence in one’s ability to consume healthy foods [53,54]. Furthermore, health-conscious consumers tend to feel more capable of making healthy food choices because they are knowledgeable about the health benefits and are motivated to act. Consequently, individuals with a strong focus on health may develop positive attitudes towards healthy foods, thereby enhancing their sense of control over consumption decisions [55,56,57]. Based on the above, the following hypotheses are proposed:

**H1.**  *Health consciousness (HC) has a positive and significant influence on self-identity (SI) in relation to healthy food consumption.*

**H2.**  *Health consciousness (HC) has a positive and significant influence on the attitude (ATT) towards consuming Andean grains.*

**H3.**  *Health consciousness (HC) has a positive and significant influence on perceived subjective norms (SN) regarding the consumption of Andean grains.*

**H4.**  *Health consciousness (HC) has a positive and significant influence on perceived behavioural control (PBC) over the consumption of Andean grains.*

Andean grains are generally perceived as expensive, which generates a substitution effect with staple foods such as rice and potatoes, or other lower-cost alternatives [58]. However, for individuals who have internalised healthy food consumption as a constitutive part of their personal identity, the price barrier is reduced [59]. Furthermore, when consumers perceive themselves as part of a health- and sustainability-oriented group, they are more willing to pay a premium price for products that reflect this self-image. In this context, purchasing decisions are not evaluated solely in economic terms, but also as an expression of coherence between personal values and self-identity [60]. For example, consumers who score highly on the Consumer Lifestyle and Health Awareness Scale (COLOHAS) are more willing to pay premium prices for sustainable products, which reinforces the connection between self-identity and the willingness to pay for Andean grains [61]. Unlike self-identity, which is evaluated from an internal motivational perspective, attitude focuses on evaluating the product from an external cognitive and affective perspective [62,63]. Therefore, attitude becomes a determining factor in the willingness to pay more for Andean grains, particularly when influenced by personal values, perceived benefits, and social factors [64].

Previous studies have shown that a positive attitude towards sustainable or nutritious foods leads to a greater willingness to pay a premium [65,66]. Specifically, for Andean grains, a positive perception of the benefits of these products could result in a high willingness to pay, meaning that consumers are more willing to pay higher prices [67]. This is consistent with the TPB theory, which identifies attitude as a key factor influencing consumer decisions, including price acceptance [49]. Similarly, subjective norms—referring to social norms and the opinions of others—may have different effects on willingness to pay, depending on whether healthy consumption is valued in a given environment [68]. Although the literature shows that social norms do not always directly impact purchase intention, the perception of control over behaviour, such as the ability to access products, can influence attitudes and ultimately purchase intention [69]. It has been demonstrated that subjective norms tend to differ depending on the context [70].

**Figure 1:**
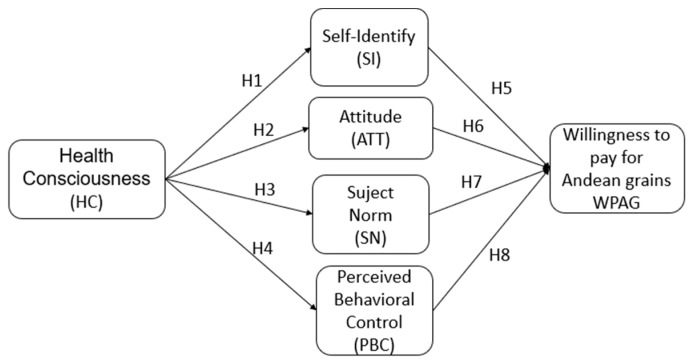
Conceptual model of health consciousness and willingness to pay for Andean grains.

Meanwhile, perceived behavioural control has emerged as the TPB component with the greatest explanatory stability in price-related decisions. This is because consumers who perceive their real purchasing capacity in economic and accessibility terms report a higher willingness to pay a premium price, particularly for products priced above conventional alternatives [54,71]. Within this framework, the willingness to pay more is understood as a decision that encompasses identity-based, evaluative, normative, and control dimensions. It is hypothesised that each construct has a positive and significant effect on this variable: health-oriented self-identity plays a key motivational role, turning the decision to pay more into an affirmation of personal coherence; attitude acts as the cognitive justification for economic expenditure; subjective norms reinforce the decision through social validation; and perceived behavioural control enables the intention to be translated into effective and sustained purchasing behaviour. Therefore, the following hypotheses are proposed (Figure 1):

**H5.**  *Self-identity (SI) has a positive and significant influence on willingness to pay for Andean grains (WPAG).*

**H6.**  *Attitude (ATT) has a positive and significant influence on willingness to pay for Andean grains (WPAG).*

**H7.**  *Subjective norms (SN) have a positive and significant influence on willingness to pay for Andean grains (WPAG).*

**H8.**  *Perceived behavioural control (PBC) has a positive and significant influence on willingness to pay for Andean grains (WPAG).*

## 3. Methodology

### 3.1. Research Design and Approach

This study took a quantitative, cross-sectional approach to examine the causal relationships between health consciousness, the components of the Theory of Planned Behaviour (TPB), and the willingness to pay for Andean grains among young, higher education consumers. This design is well-suited to testing theoretical models involving multiple latent constructs and their interrelationships [72]. The non-experimental and cross-sectional nature of the study enables perceptions and attitudes to be captured at a specific point in time without the analysed variables being manipulated.

### 3.2. Population and Sample

The target population consisted of consumers over the age of 18 residing in Lima, Peru, who had experience of purchasing or consuming food products, including Andean grains. Lima is the main consumer market in the country and is home to a socioeconomic diversity that enables representative insights into urban Peruvian consumer behaviour to be obtained. Notably, this population belongs to a consumer segment that is highly knowledgeable about the nutritional value of Andean grains. However, financial, social and time-related barriers influence their food choices. This profile is important for analysing how health awareness translates into a willingness to pay for sustainable foods in Peru, with a focus on a generation committed to sustainability and healthy eating. This aspect has received little attention in the valuation of heritage foods.

A non-probability convenience sampling technique was employed, which is commonly used in consumer behaviour research when a defined sampling frame is unavailable [72]. The final sample consisted of 600 participants, which far exceeds the minimum criteria recommended for estimating PLS-SEM models of moderate complexity. The sociodemographic data of the participants are presented in Table 1.

### 3.3. Data Collection Instrument

Data collection was carried out in the second half of 2025 using a structured, self-administered questionnaire hosted on Google Forms. The instrument included items on a five-point Likert scale (1 = Strongly Disagree; 5 = Strongly Agree), adapted from previously validated international scales and contextualised for the Peruvian setting. The constructs measured were health awareness (HA, seven items) [73] and self-identity (SI, four items), adapted from [74]. Additionally, the questionnaire adapted from [75] was used to measure aspects related to attitude (AT, 4 items), subjective norms (SN, 4 items) and perceived behavioural control (PBC, 4 items) [75]. This was used to analyse willingness to pay for Andean grains (WTP, three items), which was adapted by [11]. There is a total of 26 items. The English items were translated using back-translation and focus group testing. Prior to its final application, the questionnaire underwent expert review and a pilot test with the participation of 40 young university students to assess its clarity and comprehensibility, obtaining indicators with high internal consistency. The complete survey instrument is provided in Appendix A.

### 3.4. Statistical Analysis

The data were analysed using Partial Least Squares Structural Equation Modelling (PLS-SEM), employing WarpPLS version 8.0 software [70]. PLS-SEM is particularly suitable for exploratory and predictive models with reflective constructs and moderate sample sizes, when assumptions of multivariate normality cannot be guaranteed. WarpPLS was chosen over the covariance-based SEM (CB-SEM) approach, such as AMOS or LISREL, since CB-SEM requires large samples, strictly normal multivariate data, and well-established hypothetical models with confirmatory conclusions. Instead, the present study involves an extension of the TPB that incorporates health awareness as an external antecedent, for which a PLS-SEM exploratory–predictive structure is more appropriate. Two specific reasons were given for choosing WarpPLS. Firstly, WarpPLS incorporates a complete collinearity evaluation procedure (full VIF) that allows the simultaneous detection of vertical and lateral multicollinearity, as well as the common method bias arising from single-source survey data [70]. Secondly, WarpPLS implements algorithms capable of identifying non-linear relationships between latent constructs, which is relevant given the psychological nature of the variables examined (health awareness, self-identity, perceived behavioural control), where monotonic but non-linear effects are theoretically plausible [70]. Together, these characteristics make WarpPLS a more complete analytical tool for the current research context. The analysis was conducted in two sequential stages, in accordance with the established protocol [69].

In the first stage, the measurement model was evaluated by examining the following:

(a) The reliability of individual indicators through factor loadings (outer loadings ≥ 0.70);
(b) Internal consistency using Cronbach’s alpha and composite reliability (recommended values ≥ 0.70);
(c) Convergent validity through average variance extracted (AVE ≥ 0.50);
(d) Discriminant validity [76]. The criterion is that the square root of each construct’s AVE must exceed its correlations with other constructs. The HTMT (Heterotrait–Monotrait ratio) criterion is also used, with a conservative threshold of 0.90 [77]. Additionally, the absence of collinearity issues among constructs was verified using the full collinearity VIF index (below 5.0), as proposed by Kock [78]. In the second stage, the structural model was evaluated by analysing the path coefficients (β) and their statistical significance, using a bootstrapping resampling procedure involving 1000 subsamples. The coefficient of determination (R^2^) was also examined to evaluate the model’s explanatory power with regard to the dependent variable. Hypothesis testing was conducted at a significance level of *p* < 0.05.

## 4. Results

### 4.1. Measurement Model Assessment: Reliability, Validity, and Predictive Relevance

Table 2 shows the results of the measurement model, demonstrating adequate levels of reliability and validity for the analysed constructs. Firstly, the outer loadings of all items exceed the recommended threshold of 0.70, indicating the indicators’ adequate individual consistency and their significant contribution to their respective latent constructs [72]. These results suggest that the items adequately represent the proposed theoretical concepts.

In terms of internal reliability, Cronbach’s alpha values range from 0.882 to 0.942, and composite reliability values range from 0.919 to 0.953. Both indicators significantly exceed the recommended minimum value of 0.70, confirming the high internal consistency of the measurement scales [72,76]. Similarly, values below 0.95 usually suggest that there is no excessive redundancy among items. With regard to convergent validity, the average variance extracted (AVE) values range from 0.739 to 0.850, which exceeds the threshold of 0.50 established in the literature. This indicates that the constructs explain more than 50% of the variance of their indicators, demonstrating adequate convergence.

Finally, with regard to collinearity, full collinearity VIF values range from 1.423 to 4.132 and remain below the critical threshold of 5.0, indicating an absence of severe collinearity problems among the model constructs [78]. This suggests that the results of the structural model are not affected by multicollinearity. In terms of the model’s explanatory power, the R^2^ values for the endogenous constructs indicate that health awareness explains 21.6% of the variance in self-identity (SI), 27.1% in attitude (ATT), 17.3% in subjective norms (SN), and 28.6% in perceived behavioural control (PBC). For the final endogenous construct, the model explains 71.8% of the variance in the willingness to pay for Andean grains (WPAG), reflecting a high explanatory power [69]. Additionally, the Q^2^ Stone–Geisser coefficients confirm the predictive relevance outside the sample of all the endogenous constructs: SI (Q^2^ = 0.216), ATT (Q^2^ = 0.268), SN (Q^2^ = 0.172), PBC (Q^2^ = 0.284), and WPAG (Q^2^ = 0.719). All values are substantially above zero, with WPAG exceeding 0.50, indicating high predictive precision for the main dependent construct of the model [70].

Overall, these findings confirm that the measurement model is reliable and valid enough to proceed with evaluating the structural model (see Table 2).

### 4.2. Discriminant Validity

Table 3 shows the assessment of discriminant validity, as defined by the Fornell and Larcker (1981) [76] criterion. The results show that, in all cases, the square roots of the average variance extracted (AVE), located on the diagonal, are higher than the correlations between constructs. This indicates that each construct shares more variance with its own indicators than with other constructs in the model.

Consequently, the discriminant validity of the constructs is confirmed, demonstrating that they are empirically distinct from one another and measure conceptually different constructs. These results are consistent with the criteria recommended in the Partial Least Squares Structural Equation Modelling (PLS-SEM) literature [72].

Additionally, discriminant validity was assessed using the Heterotrait–Monotrait Ratio (HTMT) criterion, which was proposed by Christian M. Ringle and Marko Sarstedt. The results show that all HTMT values are below the conservative threshold of 0.90, indicating adequate discriminant validity among the model constructs. The SI-WPAG link requires special attention because its HTMT value (0.903) slightly exceeds the conservative threshold of 0.90; however, there are three additional pieces of evidence that corroborate the discriminant validity of these constructs. Firstly, under the Fornell–Larcker criterion, the square root of the AVE for SI (√0.819 = 0.905) and the AVE for WPAG (√0.850 = 0.922) is greater than their correlation between constructs (r = 0.83), confirming that each construct shares more variance with its own indicators than with the other construct [71]. Secondly, the conceptual distinction between SI and WPAG is well justified theoretically: SI is a cognitive construct based on identity that shows the extent to which an individual considers healthy eating as part of their self-concept, while WPAG reflects a specific economic behaviour intention, i.e., accepting a price premium for a specific product category. These constructs explain very different psychological phenomena (autoperception and economic valuation) and are measured using completely independent item sets, with no items in common. Thirdly, the high value of HTMT is theoretically expected and substantially interpretable: the theory of identity-congruent consumption and research on self-congruence consistently predict that when a product is highly aligned with the consumer’s self-identity, the predisposition to pay a high price is notably greater, precisely because the purchase makes the identity congruent. In the present study, this mechanism is corroborated by the most prominent structural route of the model (β = 0.688, *p* < 0.001), reflecting a high theoretical relationship and no redundancy in measurement. Therefore, the high value of HTMT reflects a strong substantive relationship, the same relationship that the study seeks to explain, and not an artefact of an inadequate operationalisation of the construct. Overall, these results corroborate that the constructs are empirically distinct and support the robustness of the measurement model [69] (see Table 4).

### 4.3. Structural Model Assessment

**Figure 2:**
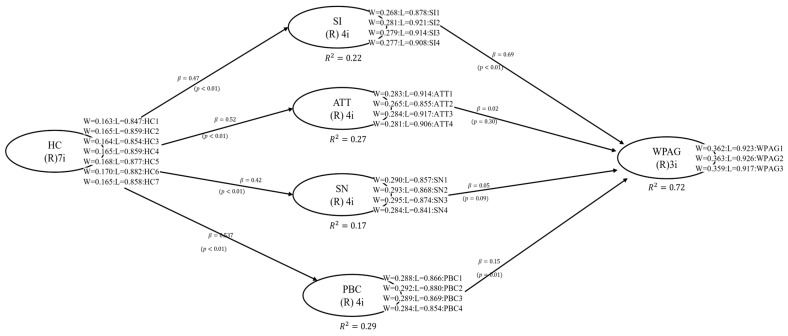
Structural model with path coefficients and R^2^ values.

Table 2 and Table 5 present the results of the hypothesis test and the structural model’s path coefficients. Health consciousness (HC) has positive and statistically significant effects on the four constructs of the TPB: perceived behavioural control (β = 0.535; t = 14.86; 95% CI [0.464, 0.606]), attitude (β = 0.520; t = 14.44; 95% CI [0.450, 0.591]), self-identity (β = 0.465; t = 12.57; 95% CI [0.393, 0.538]), and subjective norms (β = 0.416; t = 11.24; 95% CI [0.344,489), all with *p* < 0.001. The confidence intervals for the four trajectories are fully above zero, which corroborates the robustness of these effects. The effect sizes for these antecedent trajectories range from medium to medium–high (f^2^ = 0.173 to 0.286), indicating a substantially homogeneous influence of health knowledge on the cognitive and social dimensions of consumer behaviour. As shown in Figure 2, the R^2^ values for the intermediate constructs are: PBC (R^2^ = 0.29), ATT (R^2^ = 0.27), SI (R^2^ = 0.22), and SN (R^2^ = 0.17), which confirms that health awareness explains a significant proportion of the variance in each.

Regarding the prediction of the willingness to pay for Andean grains (WTP), the model explains 71.8% of its variance (R^2^ = 0.718), indicating a high explanatory power [69]. Self-identity emerges as the dominant predictor (β = 0.688; t = 19.11; 95% CI [0.618, 0.759]; *p* < 0.001; f^2^ = 0.575), with a large effect size that substantially exceeds all other predictors, confirming H5. Perceived behavioural control shows a significant but smaller contribution (β = 0.146; t = 3.84; 95% CI [0.072, 0.221]; *p* < 0.001; f^2^ = 0.097), which supports H8. In contrast, attitude (β = 0.020; t = 0.53; 95% CI [−0.054, 0.094]; *p* = 0.298; f^2^ = 0.013) and subjective norms (β = 0.051; t = 1.34; 95% CI [−0.023, 0.125]; *p* = 0.091; f^2^ = 0.034) do not reach statistical significance, with confidence intervals crossing zero and effect sizes ranging from insignificant to small, leading to the rejection of H6 and H7. The Stone–Geisser Q^2^ coefficient for WPAG (Q^2^ = 0.719 > 0.50) confirms high predictive accuracy outside the sample, validating the model’s overall predictive capacity [70]. In general, these results suggest that, in the Peruvian case, self-identity linked to healthy eating, together with perceived behavioural control, are precisely the determinants of the willingness to pay for Andean grains, but attitudes and subjective norms do not appear to be determinants. This type of pattern provides significant empirical evidence for the extension of the Theory of Planned Behaviour thanks to the introduction of health consciousness as an external antecedent and its explanatory capacity in contexts of sustainable food consumption [72,79].

Table 5 presents the results of the hypothesis testing for the structural model. It is first observed that health consciousness (HC) has a positive and statistically significant effect on self-identity (SI) (β = 0.465; *p* < 0.001), attitude (ATT) (β = 0.520; *p* < 0.001), subjective norms (SN) (β = 0.416; *p* < 0.001), and perceived behavioural control (PBC) (β = 0.535; *p* < 0.001). These results suggest that health consciousness plays a pivotal role in shaping the cognitive and social factors associated with consumer behaviour.

With regard to the willingness to pay for Andean grains (WPAG), self-identity (SI) exhibits the strongest and most significant positive effect (β = 0.688; *p* < 0.001), followed by perceived behavioural control (PBC) (β = 0.146; *p* < 0.001). Conversely, attitude (ATT) and subjective norms (SN) do not demonstrate significant effects (β = 0.020; *p* = 0.298 and β = 0.051; *p* = 0.091, respectively), thus rejecting the associated hypotheses.

Overall, these findings suggest that, in the Peruvian context, self-identity linked to healthy habits and perceived behavioural control are more decisive factors in willingness to pay than attitudes or social pressures. This provides relevant evidence for extending the Theory of Planned Behaviour [72,79].

## 5. Discussion

Another notable finding is that self-identity has a significant impact on the willingness to pay more for Andean grains (β = 0.688; *p* < 0.001). This result reinforces the idea that consumption decisions are not only based on functional or utilitarian evaluations, but are also influenced by processes of identity construction. Values associated with health tend to become integrated into consumers’ self-concepts, promoting behaviours aligned with healthy lifestyles [80,81]. As SI has emerged as the strongest predictor of willingness to pay, paying a premium price appears to reflect not only a rational assessment of the product, but also a desire to maintain consistency between personal values and behaviour in the marketplace [82]. Unlike attitude and subjective norm, self-identity reflects a deeper, more internalised motivational mechanism. Consumers perceive the consumption of Andean grains as an integral part of their identity and how they wish to present themselves to others. This may explain why self-identity (SI) displayed greater explanatory power than attitudinal evaluations or perceived social expectations, particularly with regard to products associated with cultural heritage, health, and sustainability. In emerging markets such as Peru, where food choices are often linked to symbolic and cultural meanings, motivations related to identity may become more influential than purely cognitive assessments or external social pressure [83,84]. Therefore, one of the main theoretical contributions of this study is demonstrating that identity-based mechanisms can surpass traditional TPB predictors in explaining premium purchasing behaviour in sustainable food contexts.

A notable finding is that, although health concerns (HC) significantly influenced attitude and subjective norm, these variables did not significantly affect willingness to pay a premium (WPAG) (β = 0.020; *p* = 0.298; β = 0.051; *p* = 0.091). This contrasts with traditional TPB assumptions, in which attitude and subjective norm often play central roles in predicting willingness to pay more for a product [83,84]. Nevertheless, the results align with studies suggesting that the impact of these constructs may be limited or indirect, depending on the cultural context or the extent to which consumers are familiar with the product [85,86]. The non-significant effects may also be associated with the characteristics of the sample, which was mainly composed of young university students who may already hold relatively homogeneous positive attitudes towards healthy and sustainable foods. This reduces the explanatory variability of these constructs. Similarly, Andean grains are a culturally familiar product category in Peru, which could diminish the influence of external social approval on purchasing decisions. Another possible explanation is that the willingness to pay a premium involves a stronger economic commitment that cannot be fully explained by favourable attitudes or normative perceptions alone [87,88]. Therefore, although consumers may recognise the benefits and social desirability of Andean grains, this may not necessarily result in them being willing to pay more.

When contrasted with the stronger role of self-identity, these findings suggest that, in contexts of sustainable food consumption, cognitive evaluations and perceived social pressure may lose explanatory power relative to more internalised motivational drivers. Recent evidence has also shown that health consciousness can indirectly influence behaviour through perceived norms, without necessarily resulting in a willingness to pay more for a specific product [9,70]. In the Peruvian context, attitude and subjective norm may function as background conditions, but are not sufficient triggers for concrete economic commitments, such as paying a premium for Andean grains [87,88].

Furthermore, this research demonstrated that perceived behavioural control (PBC) has a positive and significant effect on willingness to pay more (β = 0.146; *p* < 0.001). This indicates that perceived accessibility and economic capability remain relevant factors in consumer decision-making. This finding aligns with previous studies that emphasise the importance of facilitating conditions for the adoption of sustainable behaviours [89,90]. Previous research has also shown that being health-conscious strengthens the perception of control and encourages healthier choices [91]. However, in the Peruvian socioeconomic context, consumers frequently face structural barriers such as high prices, unequal access to healthy foods, and limited availability of specialised products in certain urban and rural areas. Under these conditions, perceived behavioural control becomes especially relevant, as consumers are more likely to pay a premium when they believe they have the financial resources, product availability, and knowledge to make these purchases in the long term. Therefore, when consumers perceive Andean grains as beneficial and believe they can access and afford them despite market barriers, they are more willing to pay a higher price. Overall, the findings suggest that food-related purchasing behaviour is shaped by the interaction of personal beliefs, identity processes, and perceptions of control. In the case of culturally meaningful, health-oriented products such as Andean grains, identity-based motivations appear to be a stronger determinant of premium purchasing decisions than traditional attitudinal or normative mechanisms.

## 6. Conclusions

This study tested eight hypotheses, six of which were accepted and two were rejected. Through a survey of 600 consumers and the use of PLS-SEM, findings of high theoretical and practical relevance were obtained. With regard to health consciousness, it was found to influence the components of the Theory of Planned Behaviour (TPB) among Peruvian consumers. This means that consumers with a greater awareness of health tend to develop a stronger sense of identity as healthy consumers. This makes them more likely to have favourable attitudes towards Andean grains, be more sensitive to social norms, and feel more in control of their purchasing behaviour. These results validate the theoretical framework of this research, which proposes that health consciousness strongly influences the food choice process.

Regarding the predictive power of the WPAG model, self-identity was the most powerful predictor, with a β value of 0.688. This was followed by perceived behavioural control (β = 0.146). In contrast, attitude and subjective norms were not direct predictors. In the Peruvian context, this implies that the decision to pay a premium price for Andean grains is driven by identity congruence. In other words, consumers’ self-concept as health-conscious individuals is reflected in their consumption of Andean grains and their confidence in their ability to incorporate these products into their daily diet.

Furthermore, the findings contribute in three ways. Firstly, they extend the application of the Theory of Planned Behaviour (TPB) to the study of WPAG for traditional and sustainable food products in a Peruvian context. This demonstrates the TPB’s explanatory power in both developing and high-income country settings, as evidenced by the literature review. Secondly, self-identity was identified as a mechanism through which health consciousness (HC) influences the willingness to pay a premium (WPAG) for Andean grains. Thirdly, the study provides evidence that health concerns can promote sustainable consumption in emerging urban markets.

In summary, this research provides evidence that health awareness is a strong predictor of sustainable eating behaviour, the influence of which is mediated by the components of the TPB. However, not all of these components carry the same weight when deciding to purchase Andean grains. For example, self-identity had an effect size of β = 0.688, whereas perceived behavioural control had an effect size of β = 0.146. In this case, although both self-identity and perceived behavioural control are activated by health consciousness, they do not directly translate into economic commitment. These findings suggest that promoting the sustainable consumption of Andean grains requires interventions that strengthen consumers’ self-identity and perceived ability to access these products simultaneously, rather than strategies focused solely on informing or persuading.

### 6.1. Limitations and Future Research

This study makes a significant contribution to this area of research, but it also has limitations that could be explored in future studies. Firstly, the geographical scope of the sample is limited to Lima, Peru’s urban centre, which is characterised by specific sociodemographic, cultural, and commercial profiles. Consequently, the results may not be representative of other Peruvian cities or rural areas. Future studies could therefore replicate the model in other Peruvian cities, such as Cusco, Puno, or Arequipa, or conduct transnational comparisons with neighbouring countries, such as Bolivia and Ecuador. The aim would be to evaluate the moderating role of cultural proximity to Andean grains.

Secondly, this study is based entirely on self-reported data collected using a cross-sectional design. This methodological choice introduces a risk of bias and limits the ability to establish causal relationships. Furthermore, self-reported willingness to pay may differ from actual purchasing behaviour. Therefore, future studies could benefit from combining survey data with transactional records from retail platforms or cooperative markets, and from adopting longitudinal or experimental designs that allow the causal relationship between health consciousness (HC), self-identity, and willingness to pay (WTP) to be determined.

Thirdly, the lack of significance of attitudes and subjective norms as direct predictors of willingness to pay calls for more in-depth theoretical research. Although these constructs were activated by HC (H2 and H3), this did not translate into WTP. Future research should therefore examine whether these constructs act as partial mediators or contextual moderators for the consumption of Andean grains. Analysing their mediating capacity could clarify the mechanisms through which these components of the TCP contribute, or fail to contribute, to economic commitment to sustainable food products.

Fourthly, the model is limited to Andean grains as a product category. While this allows for a precise contextual analysis, the findings cannot be generalised to other traditional and sustainable foods. Therefore, future research could extend beyond Andean grains to include other sustainable, health-promoting products, such as native tubers, traditional legumes, and indigenous Amazonian foods. This would allow us to determine whether the HC → self-identity → WPAG pathway is a generalisable pattern within traditional Peruvian and Latin American food systems as a whole.

### 6.2. Implications

The findings have significant implications for stakeholders involved in promoting and marketing sustainable foods. Health organisations should encourage healthy behaviours among consumers by implementing policies that integrate nutrition education into academic settings, public health initiatives, and other food-related contexts. This will be an effective way of driving behavioural change. For example, universities and public institutions could implement educational workshops, healthy eating campaigns, and community programmes that promote the nutritional and cultural benefits of Andean grains. Incorporating these products into school and university meal programmes could also increase familiarity with them and normalise their consumption among younger consumers.

From a marketing perspective, particularly in marketing and brand management, efforts should position Andean grains not only as a healthy alternative, but also as an integral part of consumers’ identities. Therefore, marketing campaigns could emphasise narratives related to cultural heritage, local pride, healthy lifestyles, and environmental responsibility. Social media strategies involving nutrition influencers, chefs, athletes, and wellness communities could further reinforce identity-based consumption patterns, particularly among younger, urban consumers.

Given the predictive power of willingness to pay, Andean grain producers and agricultural cooperatives could focus on removing access barriers. Research has shown that consumers who maintain a healthy diet and are health-conscious sometimes have limited willingness to pay due to the accessibility or cost of the product. Consequently, producers and distributors should invest in expanding distribution networks in urban retail settings and offer a diverse range of products, such as pre-cooked items, flours, and ready-to-eat products, while ensuring affordable access through viable marketing models. Specific interventions could include forming strategic alliances with supermarkets, local markets, and e-commerce platforms to improve product availability, as well as implementing promotional pricing strategies, discount programmes, and smaller package formats to make products more affordable for lower-income consumers.

For sustainability and environmental organisations, the link between healthy eating and sustainable food choices reinforces the argument for health-focused communication as a means of encouraging environmentally friendly consumption. Andean grains are nutritionally superior, ecologically resilient, and culturally significant. Therefore, launching a campaign to raise awareness of these three values (health, the environment, and cultural identity) among urban consumers could increase the impact of health consciousness on sustainable purchasing intentions, thereby contributing to the broader goal of achieving a sustainable food system. Additionally, collaborative initiatives involving government agencies, NGOs, and local producers could promote certification labels, sustainable food fairs, and urban awareness campaigns that emphasise the environmental resilience and cultural significance of Andean grains in Peruvian cuisine.

## Figures and Tables

**Table 1:** Sociodemographic data of the participants (N = 600).

Categories	Age Range (Years)	Frequency (*n*)	Percentage (%)
Gender	Male	268	44.70%
	Female	332	55.30%
Age range	18–25	511	85.17%
	26–35	77	12.83%
	36–45	10	1.67%
	46 and above	2	0.33%
	Total	600	100%

**Table 2:** Measurement model results: indicator loadings, predictive relevance (R^2^, Q^2^), internal consistency, convergent validity, and full collinearity assessment.

Variable	CODE	Loading	R^2^	Q^2^	α	CR	AVE	VIF
Health consciousness (HC).	HC1	0.847	-	-	0.942	0.953	0.744	1.423
	HC2	0.859						
	HC3	0.854						
	HC4	0.859						
	HC5	0.877						
	HC6	0.882						
	HC7	0.858						
Self-identify (SI):	SI1	0.878	0.216	0.216	0.926	0.948	0.819	3.741
	SI2	0.921						
	SI3	0.914						
	SI4	0.908						
Attitude (ATT)	ATT1	0.914	0.271	0.268	0.92	0.944	0.807	3.212
	ATT2	0.855						
	ATT3	0.917						
	ATT4	0.906						
Subject: norms (SN)	SN1	0.857	0.173	0.172	0.882	0.919	0.739	2.689
	SN2	0.868						
	SN3	0.874						
	SN4	0.841						
Perceived behavioural control (PBC)	PBC1	0.866	0.286	0.284	0.89	0.924	0.752	4.132
	PBC2	0.88						
	PBC3	0.869						
	PBC4	0.854						
Willingness to pay for Andean grains (WPAG):	WPAG1	0.923	0.718	0.719	0.912	0.945	0.85	3.552
	WPAG2	0.926						
	WPAG3	0.917						

Note: CR = composite reliability; AVE = average variance extracted; VIF = Full Collinearity Variance Inflation Factor; α = Cronbach’s Alpha; R^2^ = coefficient of determination; Q^2^ = Stone–Geisser coefficient. Threshold criteria follow Hair et al. [69] and Kock [70]: CR ≥ 0.70; AVE ≥ 0.50; VIF ≤ 5.0; Q^2^ > 0. HC is an exogenous construct; R^2^ and Q^2^ are not applicable (-).

**Table 3:** Discriminant validity, as measured by the Fornell–Larcker criterion.

	HC	SI	ATT	SN	PBC	WPAG
HC	0.862					
SI	0.448	0.905				
ATT	0.482	0.614	0.899			
SN	0.372	0.694	0.659	0.86		
PBC	0.502	0.659	0.82	0.738	0.867	
WPAG	0.455	0.83	0.615	0.658	0.665	0.922

**Table 4:** Discriminant validity assessment using the HTMT criterion.

	HC	SI	ATT	SN	PBC	WPAG
HC						
SI	0.48					
ATT	0.516	0.666				
SN	0.408	0.768	0.732			
PBC	0.548	0.727	0.905	0.834		
WPAG	0.491	0.903	0.671	0.734	0.738	

**Table 5:** Structural model results and hypothesis testing.

H	Path	β	*t*-Statistic	95% CI [LL, UL]	*p*-Value	Decision
H1	HC → SI	0.465	12.57	[0.393, 0.538]	<0.001	Accepted
H2	HC → ATT	0.52	14.44	[0.450, 0.591]	<0.001	Accepted
H3	HC → SN	0.416	11.24	[0.344, 0.489]	<0.001	Accepted
H4	HC → PBC	0.535	14.86	[0.464, 0.606]	<0.001	Accepted
H5	SI → WPAG	0.688	19.11	[0.618, 0.759]	<0.001	Accepted
H6	ATT → WPAG	0.02	0.53	[−0.054, 0.094]	0.298	Rejected
H7	SN → WPAG	0.051	1.34	[−0.023, 0.125]	0.091	Rejected
H8	PBC → WPAG	0.146	3.84	[0.072, 0.221]	<0.001	Accepted

## Data Availability

The data presented in this study are available on request from the corresponding author. The data are not publicly available due to privacy concerns.

## Appendix A

**Table A1:** Survey instrument in Spanish.

Conciencia de la salud	Reflexiono mucho sobre mi salud
	Soy consciente de mi salud
	Estoy alerta a los cambios en mi salud
	Asumo la responsabilidad del estado de mi salud
	Soy consciente del estado de mi salud durante el día
	Intento elegir alimentos que sean buenos para mi salud
	Prefiero productos alimenticios sin aditivos
Autoidentidad	Soy un consumidor de granos andinos
	Consumir granos andinos es parte importante de mi manera de ser
	Identificarme como consumidor de granos andinos es importante para mí
	Consumir granos andinos refleja aspectos importantes de mi identidad personal
Actitudes	Considero que comprar granos andinos es bueno
	Marco la diferencia cada vez que compro granos andinos
	Me gusta la idea de comprar granos andinos para apoyar a los productores locales
	Estoy de acuerdo que, al comprar granos andinos, contribuyo a la preservación de las tradiciones agrícolas
Normas Subjetivas	Mi familia influye en mis decisiones de compra y consumo de granos andinos
	Mis compañeros de la universidad influyen en mis decisiones de compra y/o consumo de granos andinos
	Las redes sociales influyen en mis decisiones de compra y/o consumo de granos andinos
	Pienso que la mayoría de las personas que conozco, recomiendan comprar y/o consumir granos andinos
Control Conductual Percibido	Tengo la capacidad de decidir la compra de granos andinos
	Yo puedo pagar por productos en base a granos andinos
	Me resulta fácil identificar a los granos andinos
	Los precios de los granos andinos son accesibles
Disposición a pagar por los granos andinos	Estoy dispuesto a pagar un precio más alto por los granos andinos
	Estoy dispuesto a pagar un porcentaje adicional para probar una nueva presentación en base a granos andinos
	Existe una gran probabilidad de pagar más si elijo alimentos hechos en base a granos andinos

**Table A2:** Survey instrument in English.

Health Consciousness (HC)	HC1	I think a lot about my health.
	HC2	I am aware of it.
	HC3	I am alert to changes in it.
	HC4	I take responsibility for it.
	HC5	I am mindful of my health throughout the day.
	HC6	I try to choose foods that are good for me.
	HC7	I prefer food products without additives.
Self-Identify	SI1	I consume Andean grains.
	SI2	Consuming them is an important part of who I am.
	SI3	Identifying as an Andean grain consumer is important to me.
	SI4	Eating Andean grains reflects important aspects of my personal identity.
Attitude	ATT1	I consider buying Andean grains a positive act.
	ATT2	I make a difference every time I buy them.
	ATT3	I like the idea of buying them to support local producers.
	ATT4	I agree that buying Andean grains contributes to preserving agricultural traditions.
Subjective norms	SN1	My family influences my decision to buy and consume Andean grains.
	SN2	So do my university classmates.
	SN3	Social media also influences my decisions.
	SN4	Most of the people I know recommend buying and consuming Andean grains.
Perceived Behavioural Control (PBC)	PCB1	I have the ability to choose to buy Andean grains.
	PCB2	I can afford products made with them.
	PCB3	I can easily identify Andean grains.
	PCB4	Andean grains are affordable.
Willingness to pay for Andean grains (WPAG)	WPAG1	I am willing to pay a higher price for them.
	WPAG2	I am also willing to pay a premium to try new products made with Andean grains.
	WPAG3	It is likely that I will pay more if I choose foods made with Andean grains.
